# DHEA as a Biomarker of Stress: A Systematic Review and Meta-Analysis

**DOI:** 10.3389/fpsyt.2021.688367

**Published:** 2021-07-06

**Authors:** Frédéric Dutheil, Sarah de Saint Vincent, Bruno Pereira, Jeannot Schmidt, Farès Moustafa, Morteza Charkhabi, Jean-Baptiste Bouillon-Minois, Maëlys Clinchamps

**Affiliations:** ^1^Université Clermont Auvergne, CNRS, LaPSCo, Physiological and Psychosocial Stress, CHU Clermont-Ferrand, Occupational and Environmental Medicine, Wittyfit, Clermont-Ferrand, France; ^2^Université Clermont Auvergne, Occupational and Environmental Medicine, Clermont-Ferrand, France; ^3^CHU Clermont-Ferrand, CHU Clermont-Ferrand, Clinical Research and Innovation Direction, Clermont-Ferrand, France; ^4^Université Clermont Auvergne, CNRS, LaPSCo, Physiological and Psychosocial Stress, CHU Clermont-Ferrand, Emergency Department, Clermont-Ferrand, France; ^5^Université Clermont Auvergne, CHU Clermont-Ferrand, Emergency Department, Clermont-Ferrand, France; ^6^Université Clermont Auvergne, CNRS, LaPSCo, Physiological and Psychosocial Stress, Clermont-Ferrand, France; ^7^CHU Clermont-Ferrand, CHU Clermont-Ferrand, Occupational and Environmental Medicine, Clermont-Ferrand, France

**Keywords:** stress, biomarkers, DHEA, HPA axis, psychosocial stress

## Abstract

**Background:** Psychosocial stress is a significant public health problem inducing consequences for quality of life. Results about the use of dehydroepiandrosterone (DHEA) as a biomarker of acute stress are conflicting. We conducted a systematic review and meta-analysis to demonstrate that DHEA levels could be a biomarker of stress.

**Methods:** PubMed, Cochrane Library, Embase, and ScienceDirect databases were searched on March 19, 2021 using the keywords “acute stress” AND “DHEA” OR “Dehydroepiandrosterone.” Articles needed to describe our primary outcome, i.e., induction of acute stress and at least two measures of DHEA.

**Results:** We included 14 studies, with a total of 631 participants, in our meta-analysis. The DHEA levels increased overtime after acute stress [standardized mean difference (SMD) = 1.56, 95%CI = 1.13–1.99]. Stratification by time showed a main peak at the end of stress (SMD = 2.43, 95%CI = 1.59–3.27), followed by a progressive decrease (coefficient = −0.11, 95%CI = −0.19 to −0.17, *p* = 0.020). There was no significant change 1 h after the end of acute stress. Metaregressions showed an impact of mental stress (SMD = 2.04, 95%CI = 1.43–2.65), sex (SMD = 0.02, 95%CI = 0.00–0.04), age (SMD = −0.12, 95%CI = −0.2 to −0.05), and obesity (SMD = 0.31, 95%CI = −0.00 to 0.63). There was no difference whatever the type of fluid (blood or saliva) and the measurement technique used.

**Conclusions:** DHEA is a biomarker of acute stress, with a short-term increase (1 h). DHEA increases following acute mental stress, whatever the type and duration of mental stress. Women, young people, and obese individuals had a higher response. Blood and saliva measures were comparable.

## Introduction

Psychosocial stress is a significant public health problem (1), recently increased by the coronavirus disease 2019 (COVID-19) pandemic (2). Repeated acute mental stress may also affect the quality of life and work productivity (3, 4). Identifying acute stressful events with objective measures has reached a growing interest both in physiology and preventive medicine. The physiological stress response is mediated *via* the activation of the hypothalamic–pituitary–adrenal (HPA) axis (5). Among the putative biomarkers of acute stress secreted in the adrenal cortex, dehydroepiandrosterone (DHEA) is produced by the zona reticularis area in response to adrenocorticotropic hormone (6–8). Besides being a sex steroid precursor, DHEA is an anabolic steroid with a regenerative role (9, 10). Therefore, DHEA secretion following acute stress was postulated to play a protective role as an antagonist of other stress hormones (11, 12). However, the results are conflicting about the use of DHEA as a biomarker of acute stress, despite an increasing number of publications (12–14). To our knowledge, no meta-analysis to date has examined the effects of acute stress on DHEA levels. Although dose–response relationships were never statistically assessed across the literature, some studies have reported a link between DHEA levels and stress intensity (15), whereas other studies retrieved no correlation (16). Moreover, no study has compared the influence of types of stress (mental or physical stress) on DHEA levels. Even if most of the studies have assessed DHEA levels following acute mental stress, the results seem under debate. Other factors were reported to influence DHEA responses to stress. The magnitude of change in the DHEA levels was also reported to decrease with age (14, 17, 18). Although the literature reports that DHEA response to acute stress does not differ between men and women (14), understanding sex-specific responses remains interesting. Moreover, a link has been reported between DHEA and obesity. Low serum levels of DHEA have been associated with pathological states such as obesity and high body mass index (19). DHEA administration seems to reduce body fat in normal men (20).

Therefore, we hypothesized that: (1) DHEA would be a biomarker of acute stress with an increase following acute stress; (2) there would be a dose–response relationship between the DHEA levels and characteristics of stress, such as the intensity and duration of stress or the interval between the end of stress and measures of DHEA; (3) the type of stress may influence response in DHEA levels; and 4) responses in DHEA levels after acute mental stress may be linked with other variables such as age, sex, or body mass index (BMI).

Thus, we aimed to conduct a systematic review and meta-analysis to demonstrate that DHEA levels could be a relevant biomarker of stress by summarizing all studies reporting DHEA levels in acute mental stress conditions.

## Methods

### Literature Search

**Figure 1:**
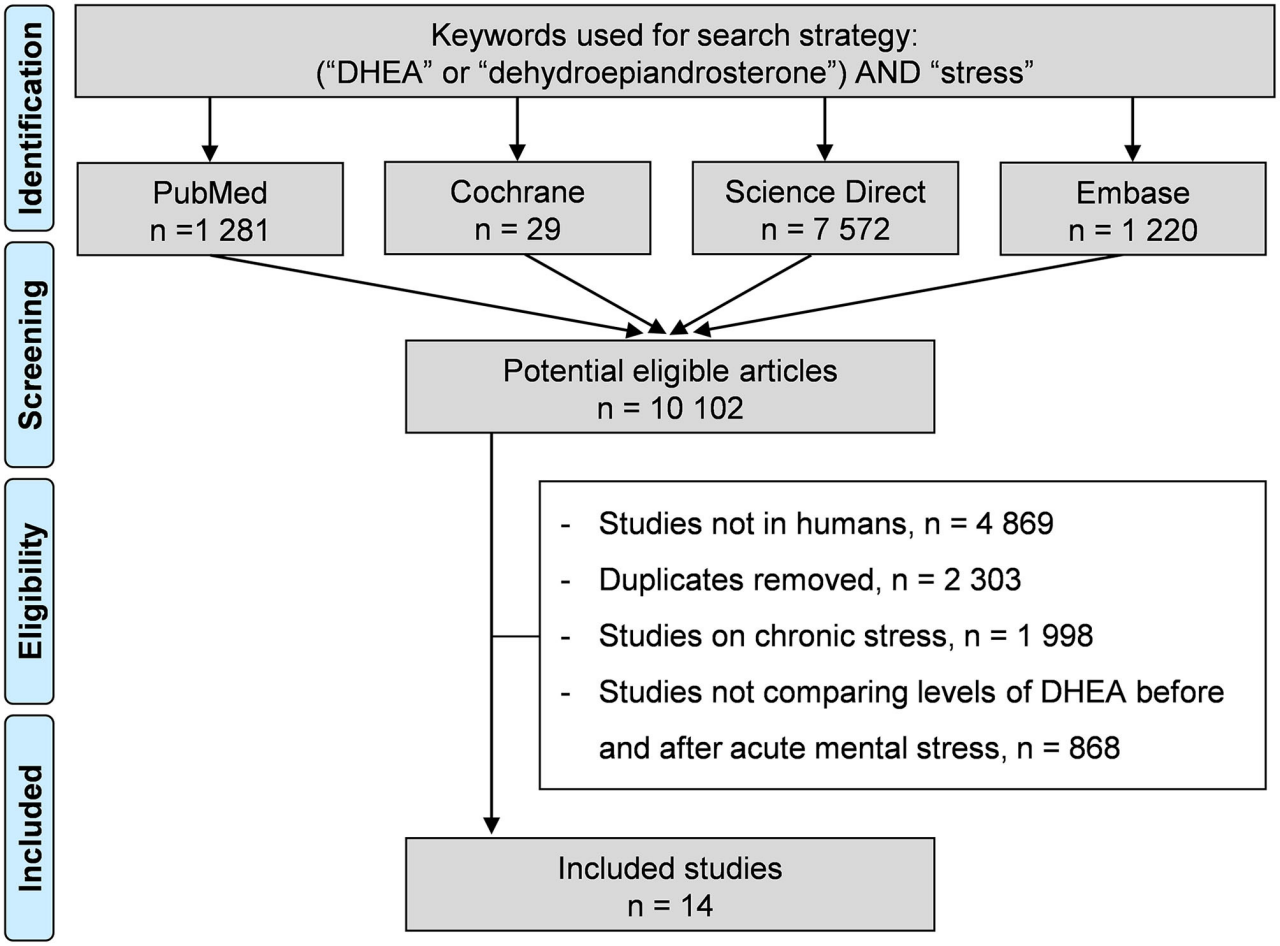
Search strategy.

We reviewed all studies involving acute mental stress. Specifically, the search strategy's inclusion criteria were studies on humans undergoing acute stress, with a longitudinal follow-up, i.e., at least two DHEA measures (baseline and after the stress), with or without a control group. We used the following keywords: “DHEA,” or “dehydroepiandrosterone,” and “acute stress.” The following databases were searched on March 19, 2021: PubMed, Cochrane Library, ScienceDirect, and Embase. We did not limit the search to specific years, and no language restrictions applied. To be included, articles needed to describe our primary outcome variable, acute stress, and DHEA measures. Articles must have a baseline measure of DHEA, i.e., before any stress intervention. We included studies only on human subjects who underwent an experimental acute stress. When a study reported several subgroups (for example, two types of interventions), they were included in our meta-analyses. Also, the reference lists of all publications meeting the inclusion criteria were manually searched to identify any further studies not found through the electronic search. The search strategy is presented in Figure 1. Two authors (MC and SdSV) conducted all literature searches, collated and separately reviewed the abstracts, and, based on the selection criteria, decided on the suitability of the articles for inclusion. A third author (FD) was asked to review the article, where consensus on appropriateness was debated. Then, all authors reviewed the eligible articles.

### Data Collection

The data collected included the first author's name, publication year, study design, periods of studies, aims and outcomes of the included articles, sample size, characteristics of individuals (age, gender, body mass index, and smoking), the DHEA measures [levels at baseline and following acute stress, time of measures, sampling (blood, saliva, etc.), and measurement techniques (ELISA, RIA, etc.], and the characteristics of stress (type and duration).

### Outcomes

The main outcome for our meta-analysis was the change in DHEA levels following an acute stress (compared to baseline levels).

### Quality of Assessment

We used the “Scottish Intercollegiate Guidelines Network” (SIGN) checklist to assess the quality of the articles (21). For randomized controlled trials (RCTs), the 10 items identified in the SIGN checklist classified the article quality through three terms: “high quality,” “neutral,” or “low quality.” For non-RCTs, three items (1.2, 1.3, and 1.4) were not relevant, and studies cannot be rated higher than “neutral.”

### Statistical Considerations

We performed statistical analysis using Stata software (version 16, StataCorp, College Station, TX, USA) (22–30). Baseline characteristics were summarized for each study sample and reported as the mean (standard deviation) and number (percentage) for continuous and categorical variables, respectively. The increase in DHEA levels following acute stress was estimated using random effects models assuming between- and within-study variability (DerSimonian and Laird approach) (31). We described our results by calculating the effect size (ES; standardized mean differences, SMD) of the increase in DHEA levels following acute stress (31). A positive ES denoted improved performance. A scale for ES has been suggested, with 0.8 reflecting a large effect, 0.5 a moderate effect, and 0.2 a small effect (32). We first conducted a meta-analysis on DHEA levels stratified by DHEA assessment time following the end of stress: immediately after the end of stress, i.e., <1, 1–30, 31–60, and >60 min. Then, we computed meta-analysis taking into account only the first measure after the end of stress, stratified on type of stress (physical or mental), with a further stratification for acute mental stress. Lastly, we searched for potential publication bias using *I*^2^, i.e., a measure of heterogeneity between studies (considered high for *I*^2^ > 50%) and by examining the funnel plots of the aforementioned meta-analyses. To verify the strength of our results, we repeated the meta-analyses after the exclusion of studies that were not evenly distributed around the funnel base (33). We also computed several sensitivity meta-analyses depending on the type of sampling (blood, saliva, etc.) and the measurement techniques (ELISA, RIA, etc.). When possible (sufficient sample size), meta-regression was proposed to study the relationship between the prevalence and characteristics of stress or clinically relevant parameters, such as age, sex, or BMI. Type I error was fixed at 0.05.

## Results

An initial search produced a possible 10,981 articles. All articles were written in English. Removal of duplicates and articles that did not meet the inclusion criteria reduced the number to 14 included articles and 64 subgroups (13–15, 34–44) (Figure 1).

### Study Designs of the Included Articles

Among the 14 included studies, three were RCTs, 10 studies were non-RCTs, and one was non-comparative. Most studies did not have a control group without induction of acute stress (13–15, 34, 36–38, 41–44).

### Quality of Articles

**Figure 2:**
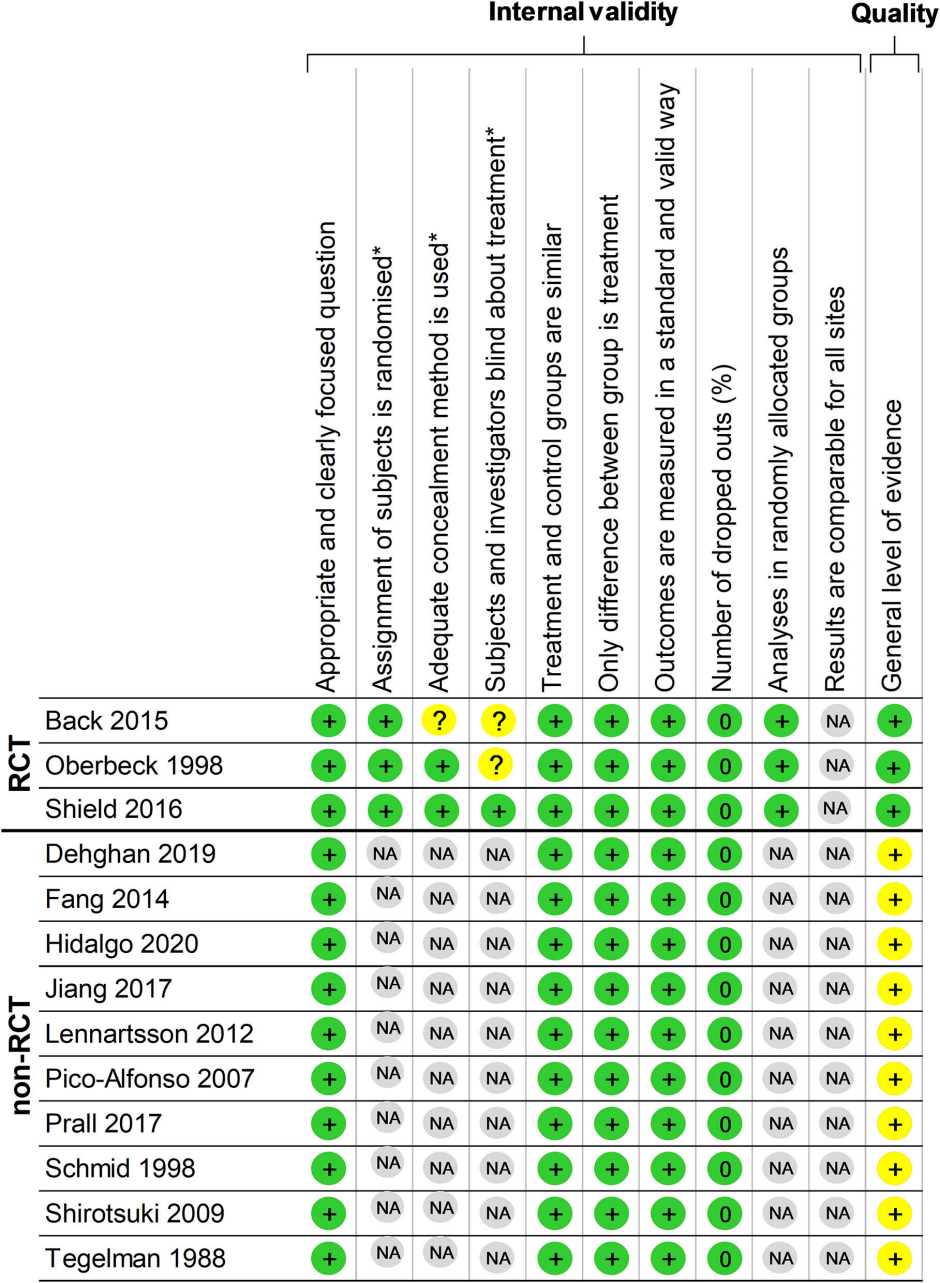
Methodological quality of the included articles using the Scottish Intercollegiate Guidelines Network (SIGN) checklist. *Items not relevant for non-randomized controlled clinical trials. ^+^Items present in the study. *Green color* represents positive impact and *yellow color* represents medium impact. *RCT*, randomized controlled trial; *non-RCT*, non randomized controlled trial; *NA*, non-applicable.

Using the SIGN checklist, the three RCTs (100%) were rated as high quality “++,” the 10 non-RCTs (100%) were rated neutral, i.e., the maximal score, and the comparative study did not require any checklist according to SIGN (45) (Figure 2). Three studies did not report any ethical approval.

### Inclusion–Exclusion Criteria Within Included Studies

DHEA measures and acute stress were the inclusion criteria of the 14 studies (Table 1). The exclusion criteria in the included studies were drug or diseases (such as Cushing's or Addison's disease, coronary heart disease, stroke, and cardiovascular disease) in seven studies (15, 35, 37, 39, 40, 42, 44), smoking in one (37), alcohol consumption in five (35–37, 42, 44), medication in nine (13–15, 35–37, 39, 40, 42, 44), to be a man in one (37) and to be a woman in another (38), irregular menstrual cycle and using oral contraceptives in one (37), BMI > 39 in one (39), to be pregnant in three (37, 39, 40), to have <30 and more than 50 years in one (15), and psychiatric disease or depression in three studies (14, 42, 44).

**Table 1:** Descriptive characteristics of the included studies.

**Study**	**Country**	**Study design**	**Characteristics of population**			**Characteristics of stress**		**Characteristics of measure**		
			**Groups**	**Men (*n*)**	**Women (*n*)**	**Type of stress**	**Duration of stress (min)**	Measurement time*	**Fluid**	**Technic**
Back et al. (39)	USA	RCT	4 groups: drug addicts + healthy stress and control in each group	38	37	Mental stress—TSST	15	1 measure: 0 min	Saliva	ELISA
Dehghan et al. (43)	Iran	Non-RCT	2 groups: elite skaters + amateur skaters	18	0	Physical stress—skate competition	120	2 measures: 0, 60 min	Saliva	ELISA
Fang et al. (15)	USA	Non-RCT	2 groups: women with high psychological response + women with low response	0	40	Mental stress—TSST	15	5 measures: 1, 15, 30, 60, 90 min	Blood	RIA
Hidalgo et al. (44)	Spain	Non-RCT	2 groups: healthy old women + healthy old men	30	35	Mental stress—TSST	10	5 measures: 15, 25, 40, 55, 70 min	Saliva	EIA
Izawa et al. (13)	Japan	Non-RCT	1 group: healthy male students	33	0	Mental stress—TSST	20	4 measures: 0, 10, 20, 30 min	Saliva	ELISA
Jiang et al. (41)	China	Non-RCT	2 groups: depressive + healthy	44	37	Mental stress—TSST	20	2 measures: 0, 50 min	Saliva	EIA
Lennartsson et al. (14)	Sweden	Non-RCT	2 groups: healthy men + healthy women	20	19	Mental stress—TSST	20	2 measures: 0, 50 min	Blood	LC-MS:MS
Oberbeck et al. (35)	Germany	RCT	3 groups: control + parachute jump with placebo + parachute jump with propanolol	24	0	Mental stress—parachute jump	6	2 measures: 0, 60 min	Blood	RIA
Pico-Alfonso et al. (37)	Italy	Non-RCT	2 groups: women in ovulatory phase + women in follicular phase	0	36	Mental stress—TSST	10	1 measure: 0 min	Blood	RIA
Prall et al. (42)	USA	Non-comparative	1 group: healthy young men	27	0	Mental stress—TSST	20	3 measures: 0, 10, 20 min	Saliva	EIA
Schmid-Ott et al. (36)	Swiss	Non-RCT	3 groups: psoriasis treated + healthy + psoriasis untreated	13	8	Mental stress—TSST	20	2 measures: 0, 60 min	Blood	RIA
Shields et al. (40)	USA	RCT	2 groups: healthy exposed to stress + healthy control	43	76	Mental stress—TSST	30	1 measure: 18 min	Saliva	ELISA
Shirotsuki et al. (38)	Japan	Non-RCT	2 groups: men with high social anxiety + men with low social anxiety	22	0	Mental stress—TSST	15	4 measures: 0, 20, 30, 40 min	Saliva	ELISA
Tegelman et al. (34)	Sweden	Non-RCT	2 groups: athletes + spectators	31	0	Physical—sport competition or mental—spectators	40	1 measure: 0 min	Blood	RIA

### Population

#### Sample Size

Population sizes ranged from 21 (36) to 119 (40). In total, we included 631 participants in our meta-analysis.

#### Gender

In total, there were 343 males (54.4%). Six studies have recruited only men (13, 34, 35, 38, 42, 43), two only women (15, 37), and six studies had mixed genders (14, 36, 39–41, 44).

#### Age

The ages of the participants ranged from 11.1 ± 1.52 (43) to 63.5 ± 0.62 years (44), with a global mean age of 30.6 ± 3.4 years.

#### Smoking

Participants were non-smokers in three studies (13, 37, 38). In another three studies, the participants were asked to not smoke before testing (14, 41, 44). In one study, the participants had a nicotine patch on the day of testing (39). Three studies (14, 15, 39) provided the smoking percentage, ranging from 11.1% (39) to 82.1% (39). Six studies did not mention the smoking status or control for smoking (34–36, 40, 42, 43).

#### Body Mass Index

Nine studies reported a BMI (13–15, 34, 37, 38, 41, 43, 44) ranging from 18.5 ± 2.5 (43) to 27.8 ± 0.7 kg/m^2^ (44). Five studies did not report BMI (35, 36, 39, 40, 42).

#### Eating, Drinking, and Hard Exercise

The participants were asked to refrain from eating, drinking, and physical exercise in four studies (13, 37, 40, 44) for 1 h before the experimental session (13), for 2 h (40, 44) or for 24 h (37).

#### Type of Population

Six studies were on healthy young individuals (13, 14, 35, 37, 40, 42), two studies were on athletes (32, 41), and one study was on healthy old individuals (44). Five studies included specific populations: one on depressive (41), one on drug addicts (39), one on people with psoriasis (36), one on high social anxiety (36), and one on high psychological responders (15). All studies included at least one subgroup of healthy individuals.

### Outcome and Aim of the Studies

The majority of the studies aimed to investigate the effect of acute stress on the HPA axis and physiological response, and more precisely on DHEA release in different populations (15, 36, 38, 39, 41). One study aimed to investigate the role of natural fluctuations of the estrogen levels (associated with different phases of the menstrual cycle) on cardiac and HPA axis activity and stress responsivity (37). All studies shared similar outcomes, i.e., variations in the biomarkers of stress following a stressful task.

### Characteristics of Acute Stress

#### Type of acute stress

All studies were in laboratory settings, i.e., 54 subgroups (13–15, 36–42, 44), except three (34, 35, 43). All laboratory studies used the Trier Social Stress Test (TSST) (46) or its derived form. The original TSST was used in eight studies (13–15, 36, 38, 41, 42, 44) and consisted of a free speech and a mental arithmetic task in front of a committee (three persons). In the study using the TSST for groups (TSST-g), the participants were in the same room (40). One used a modified TSST to answer specific questions, a mental task, and complete missing elements in a series of images (37). One used TSST and a drug cue paradigm (39). For the three studies (10 subgroups) in environmental conditions, the acute stress was a sports competition (34, 43) and a parachute jump (35).

#### Duration of Stress

All studies reported the duration of stress. The mean duration of stress was 25.8 ± 2 min across the studies, ranging from 6 (35) to 120 min (43).

### DHEA level Assessment

#### Times of Measures

All studies assessed the DHEA levels at baseline and after acute stress (13–15, 34–44). Most studies assessed DHEA levels at least twice after the end of acute stress, except for two studies that reported only DHEA once after acute stress (34, 39). After the end of stress, the follow-up duration ranged from 0 (34, 39) to 95 min (44). Baseline measure was realized at the beginning of stress in all the studies, except when the baseline measure was realized 234 min before the beginning of stress (35). The second measures were realized directly after stress in the majority of studies (13, 14, 34–36, 38, 39, 41–43), except in four studies where the first measure was realized 1 (15), 10 (37), 15 (44), or 18 min (40) after the end of stress. The mean duration between baseline and the first measure after stress was 63.2 ± 1.80 min, ranging from 15 (15, 39) to 240 min (34). In total, there were 11 studies (21 subgroups) assessing DHEA levels immediately after the end of stress, seven studies (16 subgroups) between 2 and 30 min, eight studies (17 subgroups) between 31 and 60 min, and two studies (10 subgroups) after 60 min.

#### Characteristics of Sampling

The saliva sample was the most common measure to assess DHEA levels, which was used in eight studies (36 subgroups) (13, 38–44). Six studies (28 subgroups) used blood samples (14, 15, 34–37). No study assessed DHEA levels in urine.

#### Assessment Method

Five studies (24 subgroups) used the radioimmunoassay (RIA) kit from DRG-Instruments GmbH (Marburg/Lahn, Germany) (15, 34–37). Five studies (19 subgroups) used enzyme-linked immunosorbent assay (ELISA) kits from Salimetrics LLC (State College, PA, USA) (13, 38–40, 43). One study (four subgroups) used liquid chromatography coupled to tandem mass spectrometry (LC-MS/MS) (14). Three studies (17 subgroups) used enzyme immunoassay (EIA) kits from Salimetrics (Suffolk, UK) (41, 42, 44). The results were given in nanomoles per liter in seven studies (13, 14, 34, 38, 41, 43, 44), in picograms per milliliter in three studies (40, 42, 46), nanograms per milliliter in three studies (15, 35, 36), and in micrograms per milliliter in one study (37).

### Meta-Analysis on DHEA Levels Following an Acute Stress

**Figure 3:**
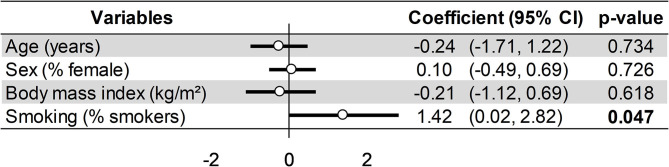
Meta-analysis on dehydroepiandrosterone (DHEA) changes following acute stress stratified by time after stress.

**Figure 4:**
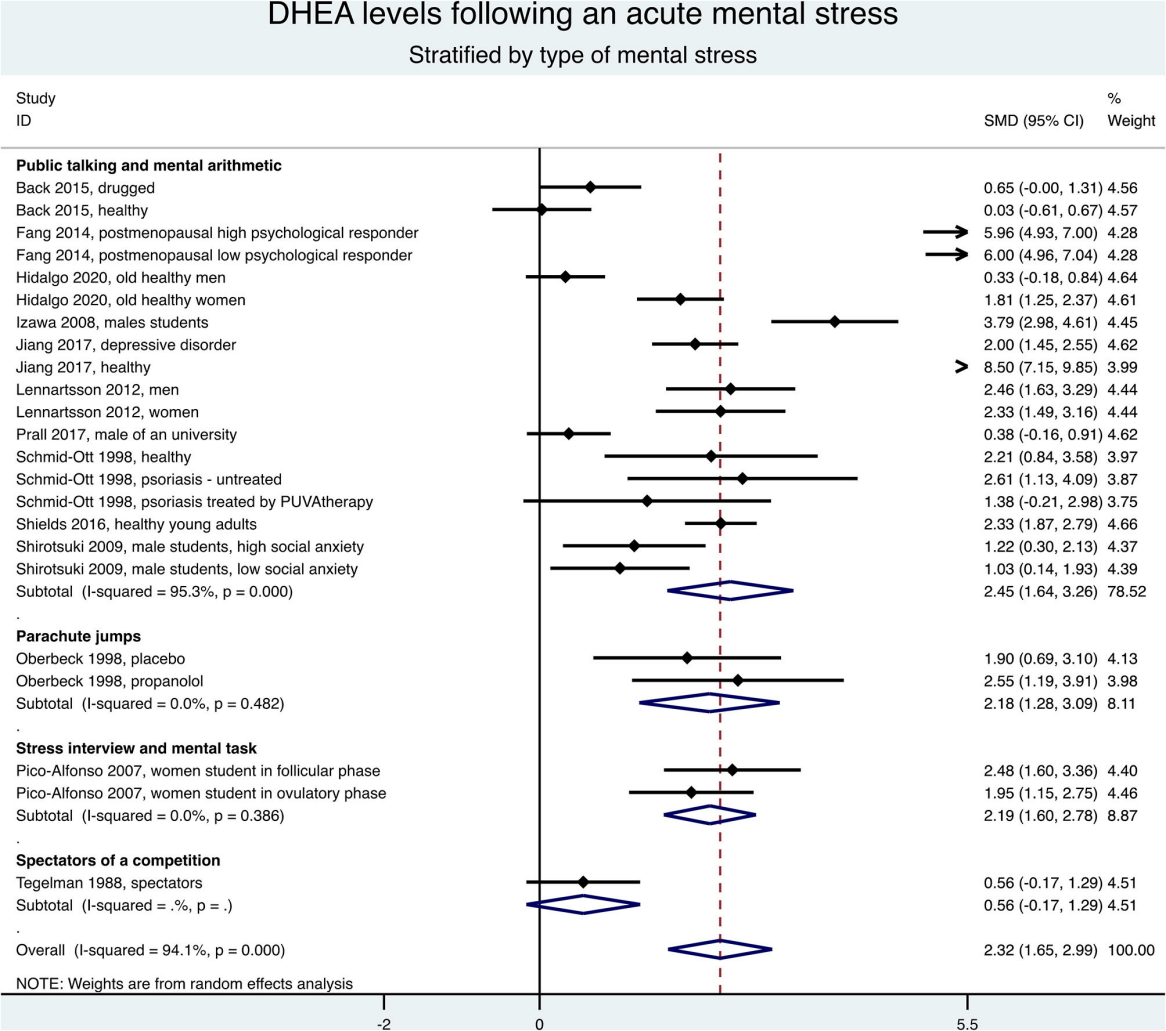
Meta-regression on factors influencing dehydroepiandrosterone (DHEA) changes following an acute stress.

The DHEA levels increased globally over time after acute stress (SMD = 1.56, 95%CI = 1.13–1.99, *p* < 0.001, *I*^2^ = 95.6%), with a main increase immediately after the end of stress (SMD = 2.43, 95%CI = 1.59–3.27, *p* < 0.001, *I*^2^ = 94.7%) compared to baseline, followed by a progressive decrease: between 2 and 30 min (SMD = 2.14, 95%CI = 1.43–2.84, *p* < 0.001, *I*^2^ = 94.1%), between 31 and 60 min (SMD = 0.89, 95%CI = 0.12–1.66, *p* = 0.023, *I*^2^ = 93.8%), and after 60 min (SMD = 0.06, 95%CI = −0.91 to 1.03, *p* = 0.901, *I*^2^ = 97%; Figure 3). Metaregressions confirmed a significant linear decrease of the DHEA levels (SMD = −0.11, 95%CI = −0.19 to −0.17, *p* = 0.020) following the initial peak of DHEA at the end of stress (Figure 4).

### Meta-Analysis Stratified by Type of Stress

**Figure 5:**
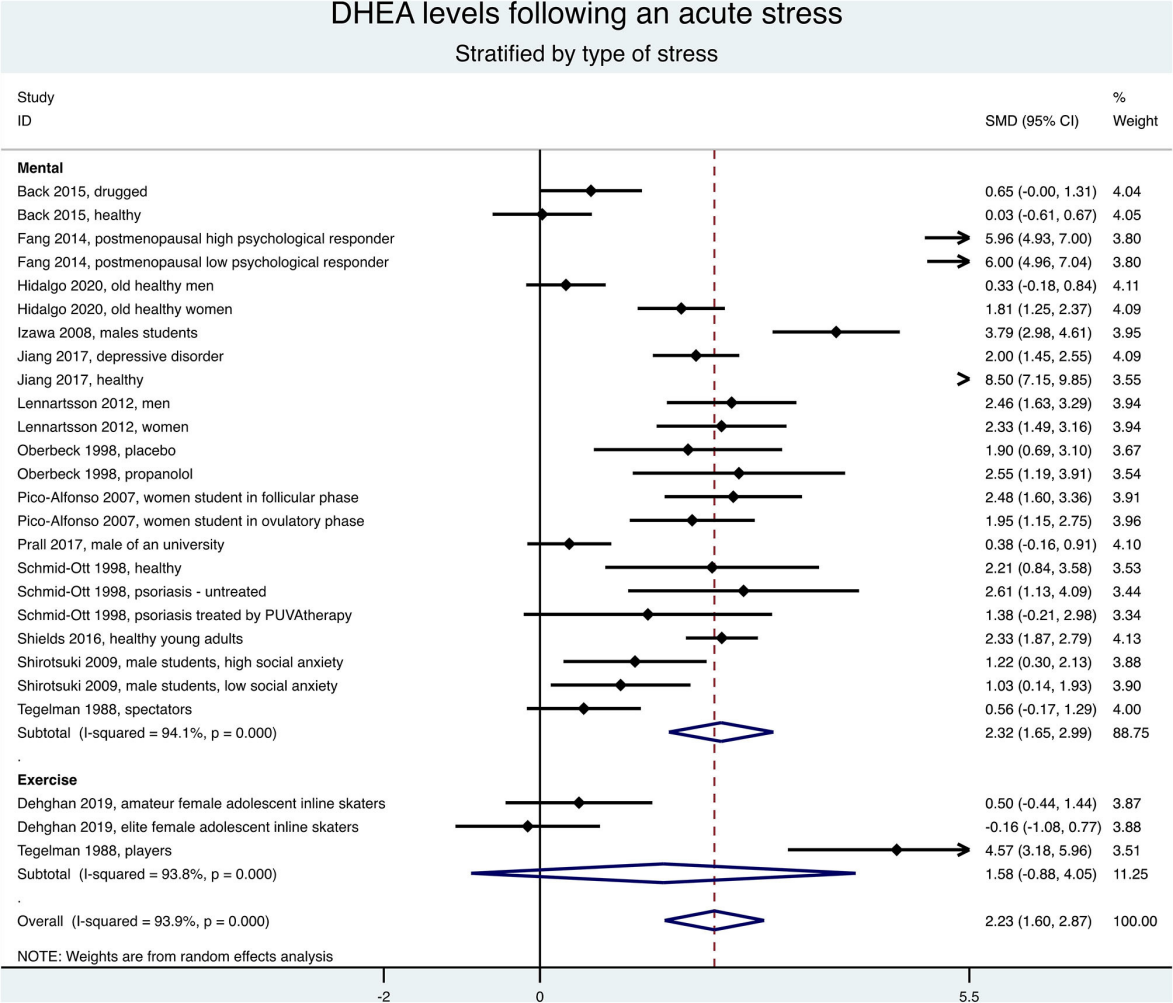
Meta-analysis on dehydroepiandrosterone (DHEA) changes between the first measure after the end of stress and baseline, stratified by type of acute stress (mental or exercise).

**Figure 6:**
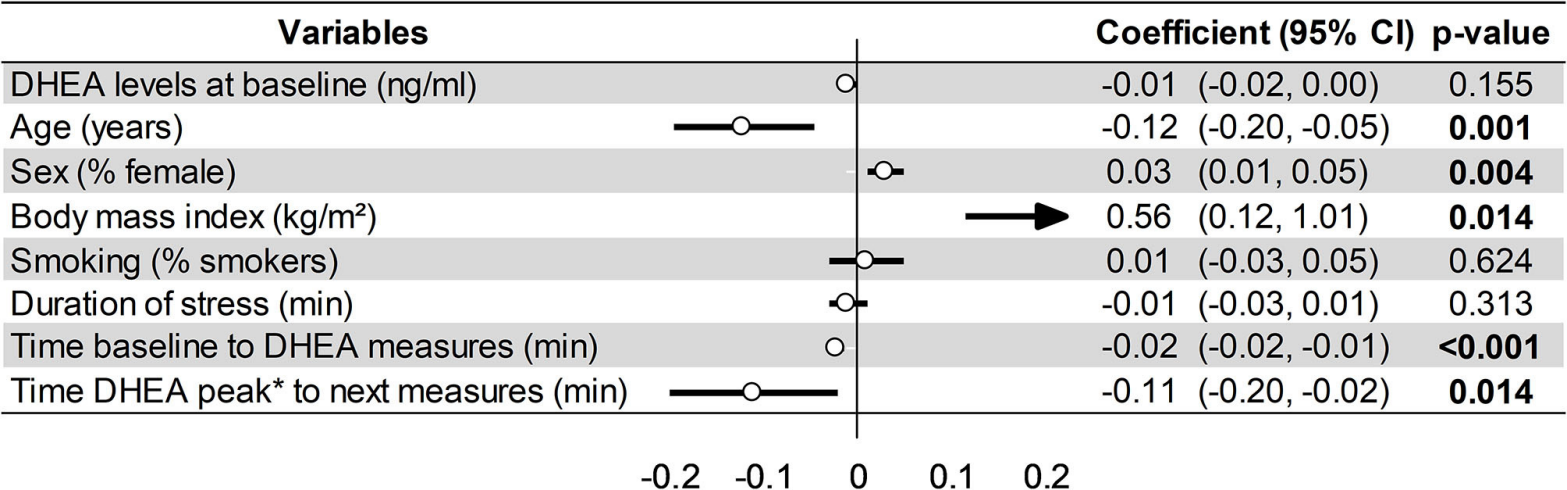
Meta-analysis on dehydroepiandrosterone (DHEA) changes between the first measure after the end of stress and baseline, stratified by type of acute mental stress.

Keeping only the first measure after the end of stress, the meta-analysis stratified by type of stress demonstrated an overall increase of the DHEA levels (SMD = 2.23, 95%CI = 1.60–2.87, *p* < 0.001, *I*^2^ = 93.9%), especially for mental stress (SMD = 2.32, 95%CI = 1.65–2.99, *p* < 0.001, *I*^2^ = 94.1%) (Figure 5). Stratification by type of acute mental stress showed an increase of DHEA levels after a public speaking and mental arithmetic (SMD = 2.45, 95%CI = 1.64–3.26, *p* < 0.001, *I*^2^ = 95.3%), a stress interview and a mental task (SMD = 2.19, 95%CI = 1.60–2.78, *p* < 0.001, *I*^2^ = 0%), or a parachute jump (SMD = 2.18, 95%CI = 1.28–3.09, *p* < 0.001, *I*^2^ = 0%; Figure 6). The results were similar when considering all times of measures after the end of stress (data not shown) and the exclusion of studies not evenly distributed around the metafunnel (Supplementary Figure 1).

### Factors Influencing DHEA Levels and Sensitivity Analyses

**Figure 7:**
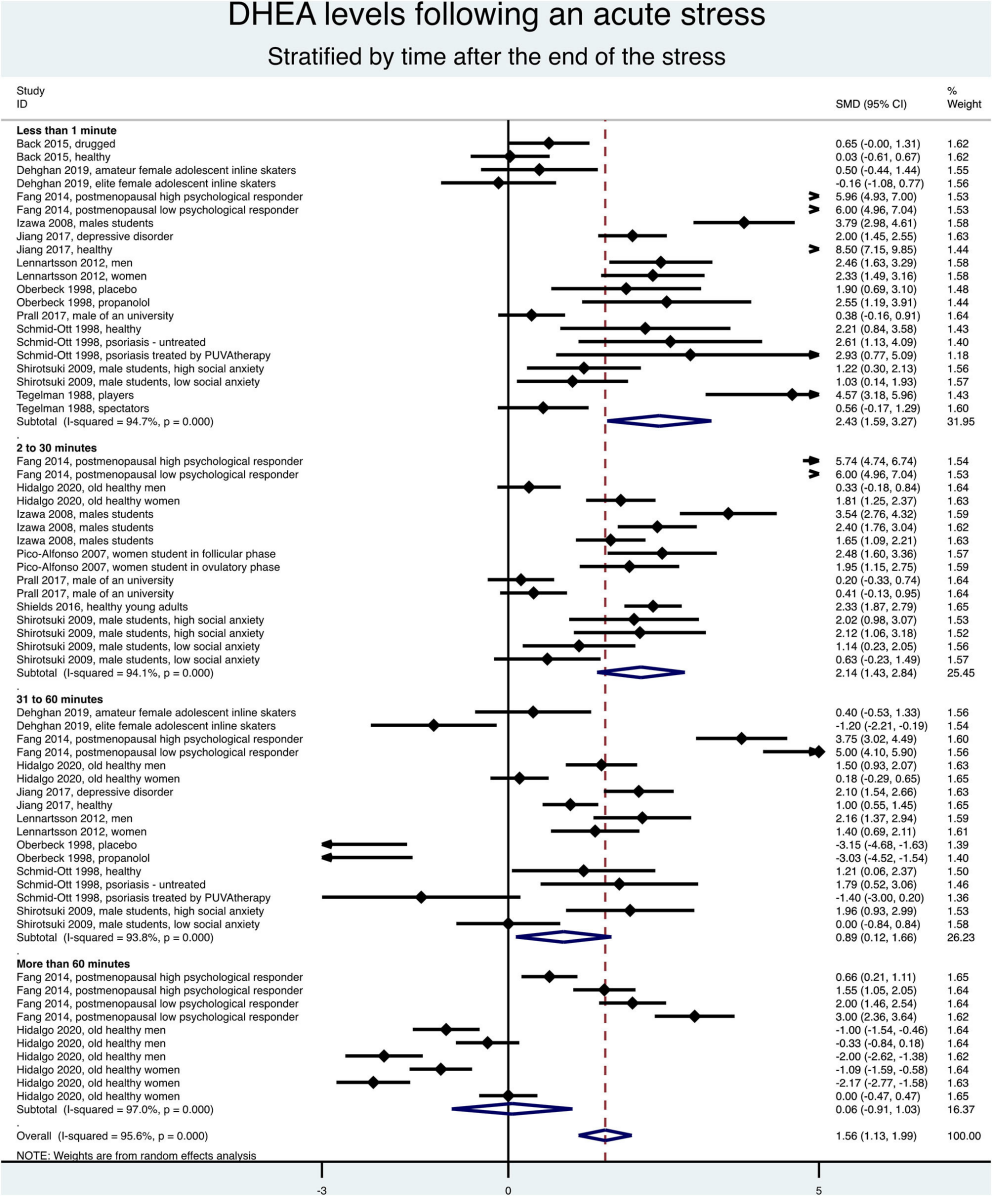
Meta-regression on factors influencing dehydroepiandrosterone (Dhea) levels at baseline (*t*_0_).

Meta-regression on factors influencing DHEA changes after the end of stress demonstrated a greater increase for young people (coefficient = −0.12, 95%CI = −0.2 to −0.05, *p* < 0.001), women (coefficient = 0.03, 95%CI = 0.01–0.05, *p* = 0.002), and those with obesity, i.e., a BMI > 30 kg/m^2^ (coefficient = 0.56, 95%CI = 0.12–1.01, *p* = 0.020). Other variables were not significantly associated (Figure 4). For the DHEA levels at baseline, smoking was the main factor responsible for high DHEA levels (coefficient = 1.42, 95%CI = 0.02–2.82, *p* = 0.047), without a significant relationship with other variables such as age, sex, or BMI (Figure 7).

Sensitivity analyses demonstrated an increase of DHEA levels after acute stress, whatever the type of fluid (blood = 2.70, 95%CI = 1.74–3.67, *p* < 0.001, *I*^2^ = 90.7%; saliva = 1.92, 95%CI = 1.02–2.82, *p* < 0.001, *I*^2^ = 95.4%) or the analysis technique (RIA = 2.77, 95%CI = 1.54–3.99, *p* < 0.001, *I*^2^ = 92.4%; EIA = 2.48, 95%CI = 0.85–4.10, *p* = 0.003, *I*^2^ = 97.2%; LC-MS/MS = 2.39, 95%CI = 1.80–2.98, *p* < 0.001, *I*^2^ = 0%; ELISA = 1.51, 95%CI = 0.44–2.57, *p* = 0.006, *I*^2^ = 93%).

## Discussion

The major finding was that DHEA is a relevant biomarker of acute stress, whatever the type of fluid (blood or saliva) and the measurement technique. DHEA demonstrated a short-term increase (1 h) following stress, with a peak at the end of stress (SMD = 2.43, 95%CI = 1.59–3.27), followed by a progressive decrease (coefficient = −0.11, 95%CI = −0.19 to −0.17). Particularly, DHEA levels increased following acute mental stress (SMD = 2.04, 95%CI = 1.43–2.65), whatever the type and duration of mental stress. Females (SMD = 0.02, 95%CI = 0.00–0.04), young people (SMD = −0.12, 95%CI = −0.2 to −0.05), and obese individuals (SMD = 0.31, 95%CI = −0.00 to 0.63) had higher increases of DHEA levels following an acute stress.

### DHEA Is a Biomarker of Stress

We demonstrated that DHEA is a biomarker of acute stress. The DHEA levels exhibited a peak at the end of stress. Then, DHEA progressively decreased and returned to baseline levels 1 h after the end of stress. This can be explained by the fact that the half-life of DHEA is short (about 30 min) (41). Very interestingly, we showed a return to the baseline after 1 h. However, some studies have found a decrease in DHEA levels at 3 months post-injury (47). We showed no difference between the type of fluid and the analysis technique, which prompted us to prefer saliva sampling. Saliva sampling is very convenient in research because it is a noninvasive method (48–51). Several studies have investigated the relationship between stress and dehydroepiandrosterone levels. DHEA has beneficial psychological effects during acute stress. It plays a protective role during the stress response, antagonizing the cortisol effects (11). Lower DHEA levels during the TSST have been related to an increase in negative mood, and reciprocally, an increase in DHEA levels following acute stress may reduce negative mood (13). Overall, there is evidence that DHEA has beneficial effects on wellbeing and cognition across the life span (52, 53). DHEA impacts the brain because it easily crosses the brain–blood barrier (44) and may be involved in preserving cortical plasticity (54). Moreover, DHEA can affect emotions, immune reactions, mood, and behavior (55).

### DHEA Levels and Characteristics of Stress

In our meta-analysis, the DHEA levels globally increased. Stratification showed the main increase after acute mental stress in comparison with acute physical stress. However, only two studies in the physical activity stratification precluded robust conclusions for physical stress. The literature is also scarce for exploring DHEA levels and physical stress, contrary to mental stress that has been extensively studied (13, 14). In our meta-analyses on types of mental stress, participants doing a parachute jump showed a high increase of DHEA levels, following literature reporting parachute jump as one of the most stressful situations (56, 57) because parachutists can face death if any problem occurs (58). Mainly, beginners in parachute jumps develop anticipatory anxiety and present higher stress responses than do experts (57). Most of the included studies in our meta-analyses used the TSST, one of the most common tools to induce acute mental stress (59, 60). We noted a high heterogeneity within the TSST stratification explained by the included population's diversity. However, the populations were too heterogeneous to perform sensitivity analyses. We can also note that the DHEAS levels increased regardless of the population studied.

### DHEA and Age, Sex, and Other Variables

We showed that younger individuals exhibited the highest increase in DHEA levels. In agreement with the literature, DHEA levels are age-dependent, with decreasing levels after early adulthood (14, 19, 53). Furthermore, the capacity to increase the DHEA levels in response to acute psychosocial stress also declines with age (14). On the other hand, in our meta-analysis, the DHEA levels were not related to age at baseline or sex. The literature is conflicting regarding sex differences for the baseline levels of DHEA. Despite some studies retrieving similar basal levels (14, 61), other studies have reported higher levels in women than in men (17, 62, 63). In our meta-analysis, women seemed to be more sensitive, with a higher DHEA increase than men. We did not find other studies reporting such a relation, with most studies comparing only the baseline levels (see above). Globally, baseline DHEA levels are age- and gender-dependent (64), and the effect of age and sex on DHEA changes following acute stress was poorly studied. We reported higher baseline levels of DHEA in smokers than in non-smokers, in agreement with the literature (65, 66). Smoking has been suggested to upregulate HPA activity (15). Finally, the main covariable influencing the increase of DHEA levels following acute stress was obesity—individuals with higher BMI experience higher DHEA response to an acute stress. This is consistent with the literature reporting the relation between obesity and DHEA response (67, 68). Although there are conflicting results on the sense of the variations, obese individuals seem to have altered responsiveness of the HPA axis (15, 69). Growing evidence suggests that modification of the gut microbiota in obesity, which can modulate inflammatory response, brain functioning, and the HPA axis (70), can explain the greater increase of DHEA levels following an acute stress. Oxidative stress may also play a role in this greater response in obese individuals (71). Moreover, very interestingly, some other biomarkers of stress have specific responses in obese compared with normal-weight individuals (72, 73).

### Limitations

Our study, however, has some limitations (74). Firstly, we did not register our methods in PROSPERO. Meta-analyses inherit the limitations of individual studies: varying qualities of the studies and multiple variations in the study protocols and evaluation. Our meta-analysis is based on a moderate number of studies, with only two studies on acute physical stress (34, 43). Despite our rigorous inclusion criteria, their quality varied. Moreover, only three studies were randomized controlled trials (33, 37, 38), precluding robust conclusions. Similarly, only three studies had a control group without stress exposure (33, 37, 38), preventing further analyses. We did not include the sulfated form of DHEA (DHEA-S)—water-soluble version of DHEA—in our meta-analysis. Indeed, DHEA-S binds more strongly to albumin than does DHEA and, consequently, has a longer biological half-life of 16 h (50). DHEA-S is a stable index of adrenocortical activity linked with chronic stress, whereas DHEA reflects the response to acute stressors (55, 75). All studies were single sites, also limiting the generalizability of our results. Although there were similarities between the inclusion criteria, they were not identical. Some studies included specific populations, such as people with depression or individuals with drug addiction, which can influence the DHEA levels (39, 41). Moreover, different methods were used to measure DHEA levels (e.g., RIA, ELISA, etc.), despite no significant differences between approaches in a sensitivity analysis. Lastly, the kind of tubes (plastic or glass) was not specified in the included studies; however, since DHEA is a relatively robust steroid, it is unlikely that the time from data collection to freezing has had any important role.

## Conclusion

We demonstrated that DHEA is a salient biomarker of acute stress with a short-term increase (1 h) following stress. More precisely, there is a peak of DHEA at the end of stress, followed by a progressive decrease. The DHEA levels increased following acute mental stress, whatever the type and duration of mental stress. Females, young people, and obese individuals had a higher increase in DHEA levels following acute stress. Blood and saliva measures were comparable, as well as the measurement techniques.

## Data Availability Statement

The original contributions presented in the study are included in the article/Supplementary Material, further inquiries can be directed to the corresponding author/s.

## Author Contributions

FD and BP conceived and designed the analysis. J-BB-M, MCl, and FD conducted the systematic literature search. J-BB-M, JS, and FM corrected the manuscript. J-BB-M, MCl, SS, and FD wrote the manuscript. J-BB-M and FD analyzed the data. SS and FD wrote the first draft of the manuscript and were responsible for the integrity of the data analysis. All authors have read and agreed to the published version of the manuscript and gave final approval for the eligibility of all articles included in the analysis and provided critical revision of the article.

## Conflict of Interest

The authors declare that the research was conducted in the absence of any commercial or financial relationships that could be construed as a potential conflict of interest.

## Supplementary Material

Supplementary Figure 1 Funnels plots analyzing for potential publication bias. SMD, Standardized Mean Difference; se (SMD), standard error of standardized mean difference. Each dot represents a single study, with its corresponding effect size (*x*-axis) and its associated standard error of the effect estimate (*y*-axis). Large, high-powered studies are placed toward the top and smaller low-powered studies toward the bottom. The plot should ideally resemble a pyramid or inverted funnel, with scatter due to sampling variation. Studies outside funnel plot are likely to present bias [Sterne JA, Sutton AJ, Ioannidis JP, Terrin N, Jones DR, Lau J, Carpenter J, Rücker G, Harbord RM, Schmid CH, Tetzlaff J, Deeks JJ, Peters J, Macaskill P, Schwarzer G, Duval S, Altman DG, Moher D, Higgins JP. Recommendations for examining and interpreting funnel plot asymmetry in meta-analyses of randomized controlled trials. *BMJ*. (2011) 343:d4002. doi: 10.1136/BMJ.d4002].
