# Supplementary figures and images for: DHEA as a Biomarker of Stress: A Systematic Review and Meta-Analysis
Source: Front Psychiatry. 2021 Jul 6;12:688367. doi: 10.3389/fpsyt.2021.688367 (PMC8290065; doi:10.3389/fpsyt.2021.688367)

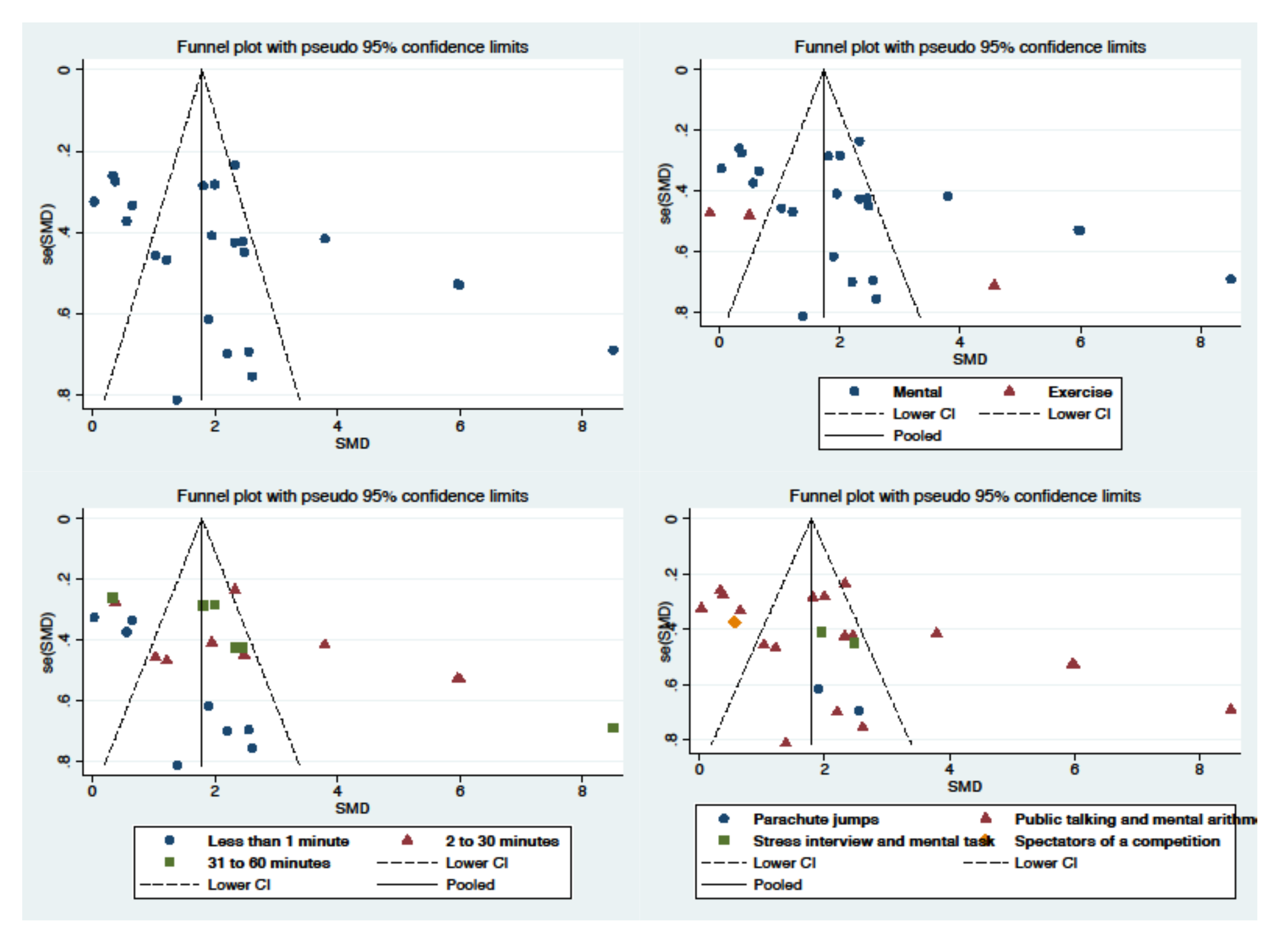

Supplement: Supplementary Figure 1 — Funnels plots analyzing for potential publication bias. SMD, Standardized Mean Difference; se (SMD), standard error of standardized mean difference. Each dot represents a single study, with its corresponding effect size (x-axis) and its associated standard error of the effect estimate (y-axis). Large, high-powered studies are placed toward the top and smaller low-powered studies toward the bottom. The plot should ideally resemble a pyramid or inverted funnel, with scatter due to sampling variation. Studies outside funnel plot are likely to present bias [Sterne JA, Sutton AJ, Ioannidis JP, Terrin N, Jones DR, Lau J, Carpenter J, Rücker G, Harbord RM, Schmid CH, Tetzlaff J, Deeks JJ, Peters J, Macaskill P, Schwarzer G, Duval S, Altman DG, Moher D, Higgins JP. Recommendations for examining and interpreting funnel plot asymmetry in meta-analyses of randomized controlled trials. BMJ. (2011) 343:d4002. doi: 10.1136/BMJ.d4002]. [file Image_1.TIF]
